# Synthesis and biological evaluation of [^131^I]iodocarvedilol as a potential radiopharmaceutical for heart imaging

**DOI:** 10.1186/s13065-023-00935-0

**Published:** 2023-03-15

**Authors:** M. A. Motaleb, K M Attalah, H A Shweeta, I. T. Ibrahim

**Affiliations:** Labeled Compounds Department, Hot Laboratories Centre, Egyptian Atomic Energy Authority (EAEA), 13759 Cairo, Egypt

**Keywords:** Carvedilol, Imaging, Iodine-131, Labelling, Heart

## Abstract

The optimization of the radiolabeling yield of carvedilol with iodine-131 was described. Dependence of the labeling yield of [^131^I]iodocarvedilol on the concentration of carvedilol, chloramine-T content, pH of the reaction mixture and reaction time was studied in details. Carvedilol was labeled with iodine-131 at pH 6 with a labeling yield of 92.6 ± 2.77% by using 100 µg carvedilol, 200 µg chloramin-T (CAT) and 30 min reaction time. The formed [^131^I]iodocarvedilol was nearly stable for a time up to one day. Biodistribution of [^131^I]iodocarvedilol was investigated in experimental animals. [^131/123^I]iodocarvedilol was located in the heart with a concentration of 19.6 ± 0.41% of the injected dose at 60 min post injection. It has a high heart uptake and heart to liver ratio, both of which are beneficial for high-quality SPECT (single-photon emission computerized tomography) myocardial imaging. [^131/123^I]iodocarvedilol solve most the drawbacks of the FDA (Food and Drug Administration) approved ^99m^Tc-sestamibi.

## Introduction

A blockage of the arteries that supply the heart muscle is known as coronary artery disease (CAD). Chest aches and breathing difficulty are signs of partial blockage, which results in lower blood supply to the heart. A complete obstruction of these veins can cause myocardial infarction by weakening and/or killing portions of the heart tissue caused by a lack of oxygen. The most common kind of heart disease and the major cause of death worldwide for both men and women is coronary artery disease (CAD).

Myocardial perfusion imaging (MPI) is a non-invasive imaging technology that utilizes an intravenously administered radiopharmaceutical to determine the distribution of blood flow in the myocardium during both rest and stress [1, 2].

The radiopharmaceutical must first reach the myocardium, where it must be up taken by live cardiac cells [3, 4].

Myocardial uptake directly relates to flow of blood, high extraction fraction, high target-to-background (T/B) ratio, good myocardial persistence (exhibiting sustained holding in the myocardium) and photon flux [5] where all of the above are desirable ideal features of a perfusion radiopharmaceutical [6].

Thallium-201 (^201^Tl) and ^99m^Tc-based myocardial perfusion tracers are used in SPECT.

The sodium-potassium adenosine triphosphatase (Na/K-ATPase) pump is responsible for ^201^Tl extraction in the heart, hence its extraction fraction is influenced by both ATPase function and blood flow [7]. Thallium-201 has a physical half-life of 73 h and emits x-rays with energies of 67–82 KeV and gamma rays with energies of 135–167 KeV.

The picture quality of thallium-201 is worse than that of technetium-labeled agents [8–10], which has limited its application [11].

^99m^Tc-Sestamibi, ^99m^Tc-tetrofosmin and ^99m^Tc-teboroxime have been approved by the FDA [12]. They all passively traverse cell membranes and have distinct myocardial accumulation and clearance characteristics. Despite having poor extraction and biodistribution qualities, due to their higher energy photons and low redistribution profiles, all three offer images of higher quality than ^201^Tl.

In nuclear cardiology, ^99m^Tc-Sestamibi has been frequently utilized for MPI. Owing to its high liver uptake [13] and roll-off at greater blood flow levels, it does not match the criteria of an ideal perfusion imaging tracer.

The high liver uptake makes evaluating cardiac activity in the inferior and left ventricular walls difficult [14].

Photon scattering from significant liver activity remains a major obstacle for successful SPECT diagnosis of heart dysfunction, despite ongoing attempts to eliminate this interference.

As a result, developing a novel perfusion radiopharmaceutical with superior biodistribution and/or extraction capabilities would be extremely beneficial [15–17].

Carvedilol is an antihypertensive drug that is used to treat elevated blood pressure and heart failure. It’s also used to boost your chances of survival after a heart attack if heart isn’t working well. Carvedilol binds to β-adrenergic receptors on cardiac myocytes in a reversible manner.

**Fig. 1:**
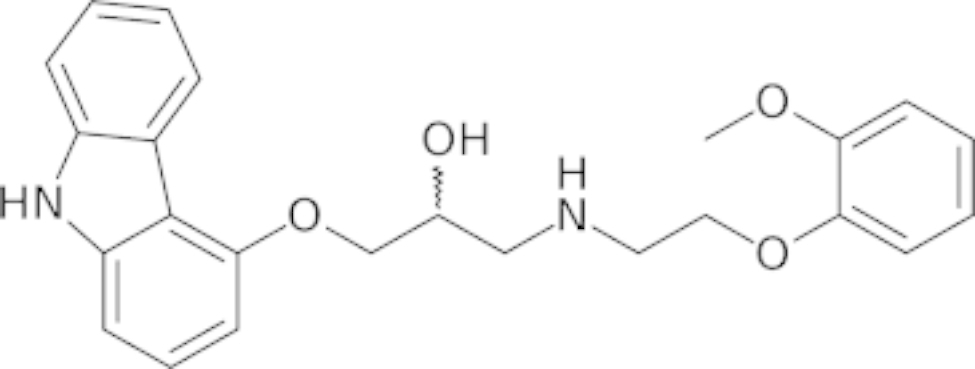
Chemical structure of carvedilol

The inhibition of these receptors inhibits the sympathetic nervous system from responding, resulting in a drop-in heart rate and contractility. This action is beneficial in heart failure patients [18] where the sympathetic nervous system is activated as a compensatory mechanism (Fig. 1) [19].

The aim of the present work was to establish a simple and efficient method for radiosynthesis, characterization and biological evaluation of [^131^I]iodocarvedilol as a potential myocardial perfusion imaging radiopharmaceutical.

## Experimental

Carvedilol was purchased from Memphis pharmaceutical company; Egypt and all other chemicals were purchased from Merck and they were analytical reagents.

### Labeling procedure

Under oxidative circumstances in the presence of CAT, [^131^I]iodocarvedilol was frequently produced via direct electrophilic substitution with no carrier added (NCA) ^131^I. NCA ^131^I allows for the use of high specific activity iodide without the need for carrier iodine.

The effect of different reaction parameters and conditions on radiolabeling efficiency, like the amount of oxidizing agents (CAT), carvedilol concentration [10 to 300 µg, (2.46 × 10^− 5^ to 7.38 × 10^− 4^ mM)], reaction pH (3–10), and reaction time (5–120 min), were studied and optimized in order to maximize radioiodination yield. No-carrier-added Na^131^I (7.2 MBq) was transferred to the reaction flask. A freshly generated CAT in methanol was added to the reaction mixture, followed by 100 µg (2.46 × 10^− 4^ mM) of carvedilol in methanol. For 15 min, the reaction mixture was agitated using a magnetic stirrer at room temperature. A drop of 10% saturated sodium thiosulfate (10 mg/mL in H_2_O) was added to breakdown the excess iodine (I_2_) and stop the reaction by reducing it to iodide (I^−^) as it oxidizes to tetrathionate (S_4_O_6_^2−^) [20], [21].

The radioiodinated product was separated, and TLC (Thin Layer Chromatography) was used to determine the radioiodination yield and purity of the product.

### Analysis

Thin-layer chromatography was used to determine the radiochemical yield and purity [22] of [^131^I]iodocarvedilol utilizing strips of silica gel coated on an alumina sheet.

1–2 µL of the reaction mixture was put 3 cm above the lower edge of a TLC strip (1.5 cm width, 15 cm length) and allowed to evaporate gradually.

HPLC (High-performance liquid chromatography) analysis: The radiochemical yield of [^131^I]iodocarvedilol was calculated by injecting 10 µL of the reaction mixture into the column Rp-18 (250 mm x 4.6 mm, 5 μm) constructed in the HPLC (Shimadzu model), which contains a set of pumps LC-9 A, Rheohydron injector (Syringe Loading Sample Injector-7125), and UV spectrophotometer detector (SPD-6 A). A mixture of acetonitrile/water (65:35) containing 0.1% trifluoroacetic acid was used as mobile phase, at a flow rate of 1.0 mL/ min. The labeled compound was collected separately by using a fraction collector up to 12 min and its activity was counted by using well a type NaI(Tl) crystal connected to a single-channel analyzer.

## Nonradioactive iodination of carvedilol

**Fig. 2:**
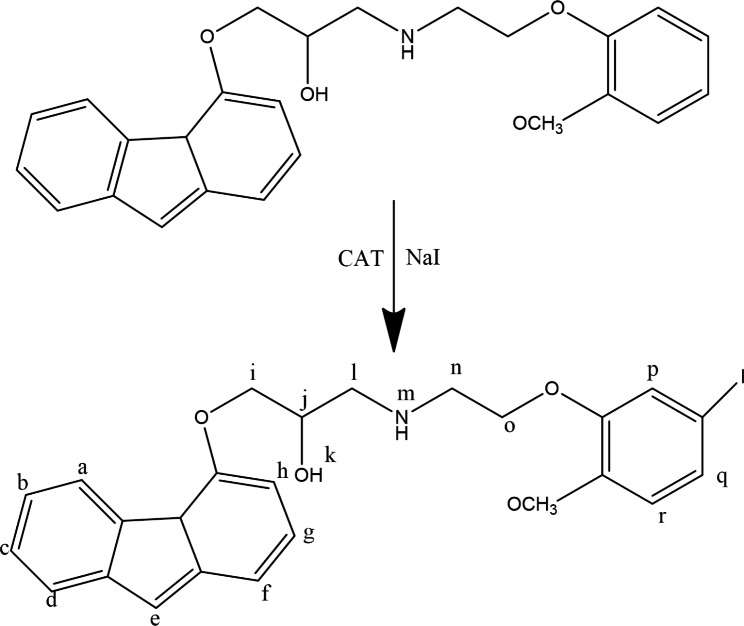
Suggested structure of ^127^I-carvedilol

Carvedilol was labeled with non-radioactive iodine with the chloramine-T method. Iodination of carvedilol generally follows the same chemistry used for radioactive iodination. Carvedilol (100 mg) was dissolved in minimum amount of methanol then a mixture of 200 µg chloramine-T and 15 mg NaI solution was added with vigorously stirring over 60 min for 1.5 h. The reaction products were separated with high performance liquid chromatography. The 1 H-NMR of ^127^ I-iodocarvedilol is similar to that of the carvedilol itself except the hydrogen in the paraposition to OCH_3_ phenyl side chain which is different. ^1^ H-NMR (δ ppm):

8.2 (d, 1 H,a indole ring CH), 7.1 (d, 1 H,b indole ring CH), 7.11 (t, 1 H,c indole ring CH), 7.25 (d, 1 H,d indole ring CH), 11.2 (s, 1 H,e indole ring NH), 6.7 (d, 1 H,f indole ring CH), 7.3 (t, 1 H,g indole ring CH), 7.45 (d, 1 H,h indole ring CH),4.15 (m,3 H, i,j 2* H*-methylene CH_2_ and 1* H*-methine CH), 5.2 (d, 1 H,k alcohol OH), 2.8 (m, 2 H,l methylene CH_2_), 2.1 (s, 1 H,m amine NH), 2.9 (t, 2 H,n methylene CH_2_), 4 (t, 2 H,o methylene CH_2_), 6.9 (s, 1 H,p 1-benzene ring), 6.8 (m, 2 H,q,r 1-benzene ring) and 3.7 (s, 3 H,t CH_3_) as shown in Fig. 2.

### In vitro stability of [^131^I]iodocarvedilol

In vitro, the stability of [^131^I]iodocarvedilol was investigated by combining 1 ml normal serum with 0.5 mL [^131^I]iodocarvedilol and incubating for 24 h at 37 ^o^C.

During the incubation, 0.2 mL aliquots were taken at varied time intervals up to 24 h and exposed to TLC to determine the percent of [^131^I]iodocarvedilol and free iodine.

As a result, the radiolabeled compound stability will decide its potential for in vivo application.

### Determination of octanol-water partition coefficient (log po/w)

Log P values of [^131^I]iodocarvedilol was determined using the following procedure: the [^131^I]iodocarvedilol was prepared and purified by HPLC. The collected mobile phases were evaporated and the residue was dissolved in a mixture of equal volume (3 mL:3 mL) n-octanol and water. After vortex for > 20 min, the mixture was centrifuged at 8,000 rpm for 5 min. Samples (in triplets) from aqueous and n-octanol were obtained and counted separately in a gamma counter. The partition coefficients were calculated using the equation: P = (activity concentration in n-octanol)/ (activity concentration in water). The log P value was measured three different times and reported as an average of three different measurements. The calculated lipophilicity equal to 1.8.

### Biological evaluation

The animal experimental protocols followed the Egyptian Atomic Energy Authority’s rules and were approved by the animal ethics committee of the Labeled Compound Department. The biodistribution of [^131^I]iodocarvedilol was studied in normal rats (purchased from Egyptian National Research Center) (n = 5) after 0.5, 1, and 3 h post injection (pi). Animals were housed in groups of five and given food and water prior to the study. Each animal received an aliquot of 10 µL containing 3.7 MBq of the purified [^131^I]iodocarvedilol via the tail vein. Each animal was housed separately before being sacrificed by cervical dislocation at 0.5, 1, and 3 h following [^131^I]iodocarvedilol injection (n = 5). Following sacrifice, each rat was weighed, and blood was collected from the heart and weighed. Muscles, bone, and blood were predicted to contribute for 40, 10, and 7% of total body weight, respectively [23]. Organs and tissues were cleaned with saline, collected in plastic containers, and weighed after dissection. In a well type NaI(Tl) well crystal linked to an SR-7 scaler ratemeter [24], the radioactivity of each sample as well as the background was quantified. For each time point, the percent injected dose (ID) per organ (percent ID/organ S.D.) in a population of five rats is presented. The Student t test was used to assess data differences. The two-tailed test results for *p* are presented, and all results are given as mean ± SEM. The level of significance was set at *p* ≤ 0.05.

## Results and discussion

**Fig. 3:**
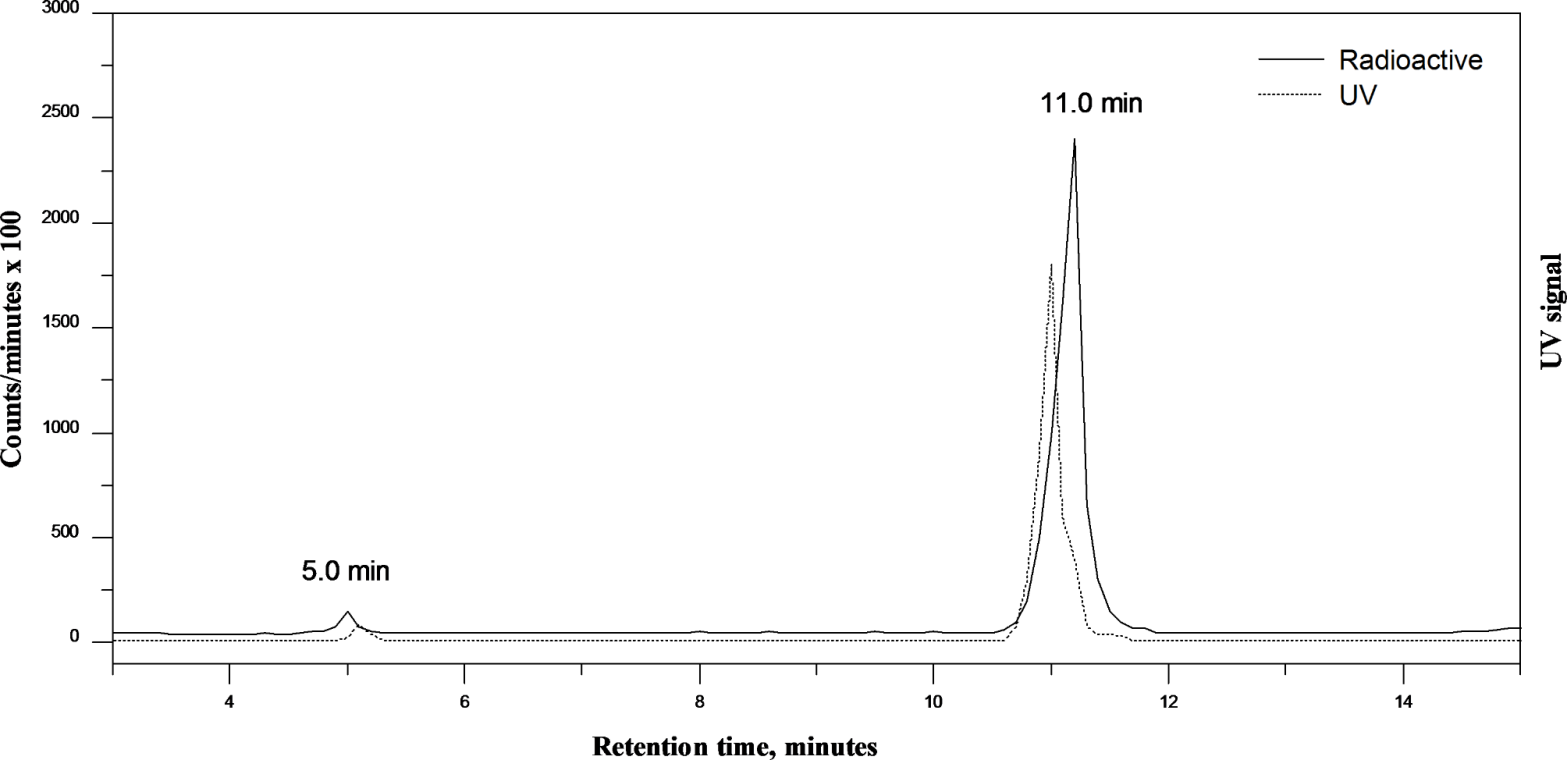
HPLC chromatogram for [^131^I]iodocarvedilol

TLC was used to determine the radiochemical purity of [^131^I]iodocarvedilol, with a developing solvent mixture of methylene chloride: ethyl acetate (2:1 v/v), where radioiodide (^131^I) remained near the origin (R_f_ = 0–0.5), while [^131^I]iodocarvedilol moved with the solvent front (R_f_ = 0.7–1). The percentage radioiodination yield was calculated as the ratio of the radioactivity of [^131^I]iodocarvedilol to the overall activity, The average of three tests is used to calculate the radioiodination yield of [^131^I]iodocarvedilol. The radiochemical purity was further confirmed by HPLC analysis, where the retention time of free iodide and [^131^I]iodocarvedilol were 5 and 11 min, respectively, as shown in the chromatogram (Fig. 3). The UV chromatogram shows a peak at 10.9, which corresponds to cold iodocarvedilol, which coincides with the [^131^I]iodocarvedilol that appeared nearly at the same retention time in the radio-chromatogram.

### Effect of reaction time

**Fig. 4:**
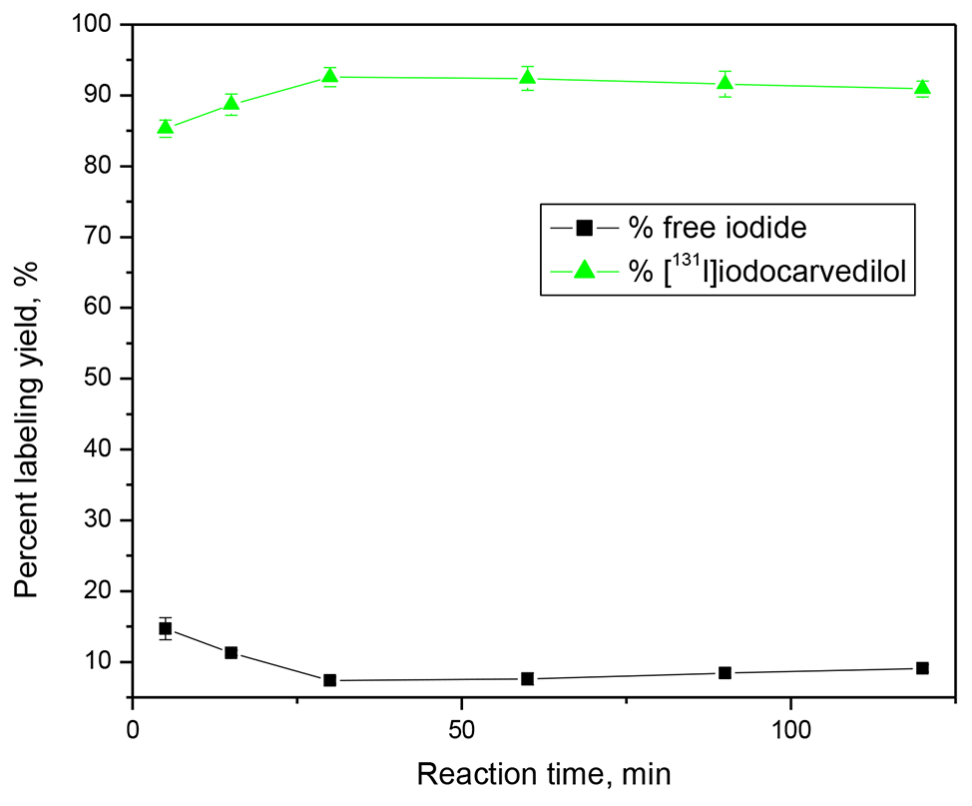
Effect of Reaction time on the radiochemical yield of [^131^I]iodocarvedilol. Reaction conditions: 100 (2.46 × 10^− 4^ mM) µg carvedilol (50 µl) + 200 µg (8.79 × 10^− 4^ mM) CAT (20 µl) + 10 µl Na^131^I for ½ hour at pH 6 and 25 °C; n = 3

The yield of labelling is highly influenced by reaction time, which might range from 5 to 120 min. Figure 4 shows that increasing the reaction time from 5 to 30 min increases the yield significantly. The radiochemical yield is unaffected by increasing the reaction time beyond 30 min. The maximal radiochemical yield (92.6 ± 2.77%) requires a minimum reaction time of 30 min.

### Effect of carvedilol amount

**Fig. 5:**
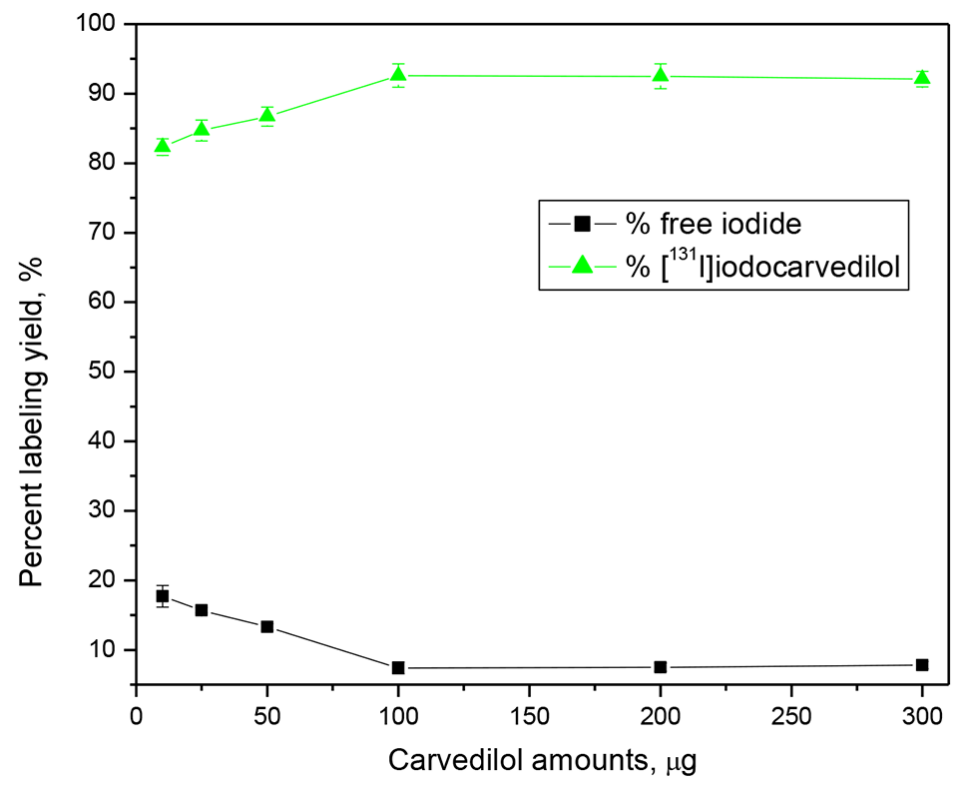
Effect of substrate amount on the radiochemical yield of [^131^I]iodocarvedilol. Reaction conditions: X µg substrate + 200 µg (8.79 × 10^− 4^ mM) CAT (20 µl) + 10 µl Na^131^I for ½ hour at pH 6 and 25 °C; n = 3

Figure 5 shows the relationship between radiochemical yield and carvedilol amount, where the radiochemical yield increased from 82.3 to 92.6% as the amount of carvedilol rose from 10 to 300 µg (2.46 × 10^− 5^ to 7.38 × 10^− 4^ mM). Further increases in the amount of carvedilol beyond 100 mg had no effect on the labelling yield, which might be explained by the fact that this concentration of carvedilol (100 µg, 2.46 × 10^− 4^ mM) is sufficient to capture the full produced iodonium ion, resulting in the highest yield (92.6 ± 2.7%).

### Effect of CAT concentration

**Fig. 6:**
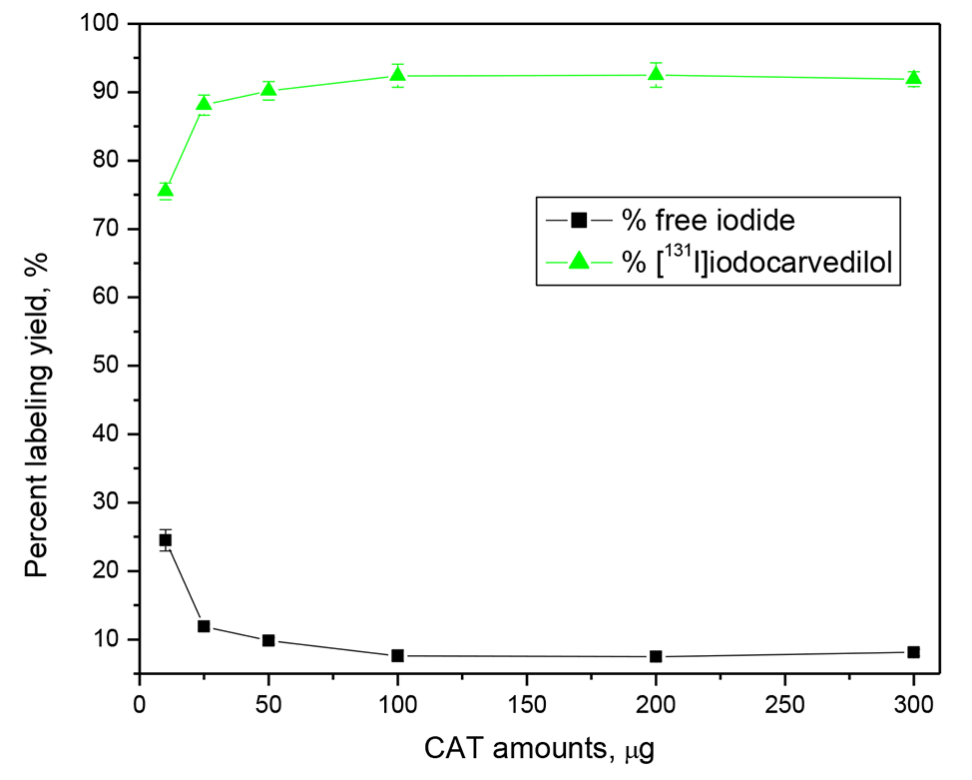
Effect of CAT content on the radiochemical yield of [^131^I]iodocarvedilol. Reaction conditions: 100 µg (2.46 × 10^− 4^ mM) substrate (50 µl) + X amounts of CAT + 10 µl Na^131^I for ½ hour at pH 6, at 25 °C; n = 3

After increasing the amount of CAT from 10 to 300 µg (4.39 × 10^− 5^ to 1.3 × 10^− 3^ mM) at pH 6 and a 30 minute reaction time, a high radiochemical yield (92.6 ± 2.7 %) was obtained. The formation of undesired oxidative by-products such chlorination, polymerization, and denaturation of Carvedilol causes a drop in iodination yield when the CAT concentration is increased over 200 µg (8.79 × 10^− 4^ mM) [25]]. The high reactivity and concentration of CAT [2] may be responsible for the production of these contaminants. As a result, the optimal concentration of CAT is strongly advised in order to avoid the occurrence of by-products and achieve high yield and purity (Fig. 6).

### Effect of pH of the reaction mixture

**Fig. 7:**
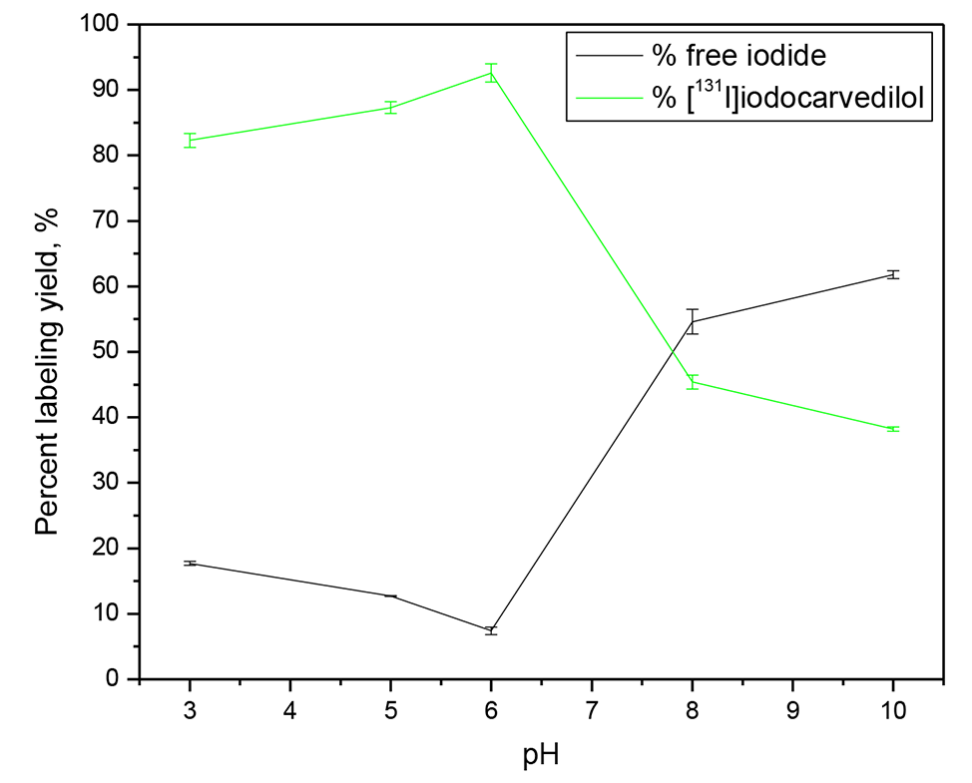
Effect of pH on the radiochemical yield of [^131^I]iodocarvedilol. Reaction conditions: 100 µg (2.46 × 10^− 4^ mM) carvedilol (50 µl) + 200 µg (8.79 × 10^− 4^ mM) CAT (20 µl) + 10 µl Na^131^I for ½ hour at different pH, at 25 °C; n = 3

Figure 7 shows the effect of the reaction mixture pH on the radioiodination yield of [^131^I]iodocarvedilol. The reaction medium’s pH was measured in the range of 3 to 10.

The redox potential of chloramine-T is pH dependent and decreases as the medium’s pH rises [26], implying that the type of active oxidizing species of CAT is influenced by the medium’s pH and reaction conditions. When chloramine-T is dissolved in water, it breaks down to ArSO_2_NCl, which is hydrolyzed in an acidic medium to produce HOCl. The hypohalous acid is hydrolyzed further to produce H_2_OCl^+^.

HOCl and H2OCl^+^ are probable oxidizing species in acidified CAT solutions, while HOCl and ClO are possible oxidizing species in alkaline CAT solutions.

Under acidic circumstances, the produced HOCl or H2OCl^+^ oxidized the iodine to the oxidative state I^+^ (iodonium) [27], which quickly reacts with any sites within carvedilol that can undergo electrophilic substitution reactions [26, 28, 29]. Due to the maximal action of CAT at almost neutral pH, the remarkable stability of the carvedilol structure, and the good protonation of the aromatic ring at this pH value, the yield was maximized (92.6 ± 2.77%), yielding H^+^, which was substituted by the active iodonium ion I^+^. The yield dropped dramatically when the pH of the reaction medium was changed to the high acidic side, reaching 82.3% at pH 3. Also, the yield was quite low on the alkaline side, reaching 45.4 and 38.3% at pH 8 and 10, respectively. The synthesis of hypoiodite ion (IO^−^) and iodate (IO_3_^−^) [30], which are not appropriate forms for radioiodination of carvedilol, could explain the decrease in radiochemical yield at alkaline pH.

### Stability test

Due to the decomposition of the [^131^I]iodocarvedilol, incubation of the preparation containing [^131^I]iodocarvedilol for 24 hours at 37 ^o^C resulted in a small decrease in the yield of [^131^I]iodocarvedilol (92.6 ± 2.77%) and the release of radioactivity from the [^131^I]iodocarvedilol was 5.9 ± 1.1%, as determined by ITLC (Table 1).

**Table 1:** *In-vitro* stability of [^131^I]iodocarvedilol

Time/hour	% Labeled compound	% Free iodide
1	92.6 ± 0.36	7.4 ± 0.1
2	91.4 ± 0.5	8.6 ± 0.15
4	88.1 ± 0.3	11.9 ± 0.2
24	86.2 ± 0.4	13.8 ± 0.1

Values represent the mean ± SEM n = 6

### In-vivo studies of [^131^I]iodocarvedilol

In compliance with the guidelines set out by the Egyptian Atomic Energy Authority, the animal Ethics Committee, Labeled Compounds Department, and the protocol approval of Research Ethics Committee in the Faculty of Pharmacy, Cairo University (REC-FOPCU), Egypt, the biodistribution studies were performed.

**Fig. 8:**
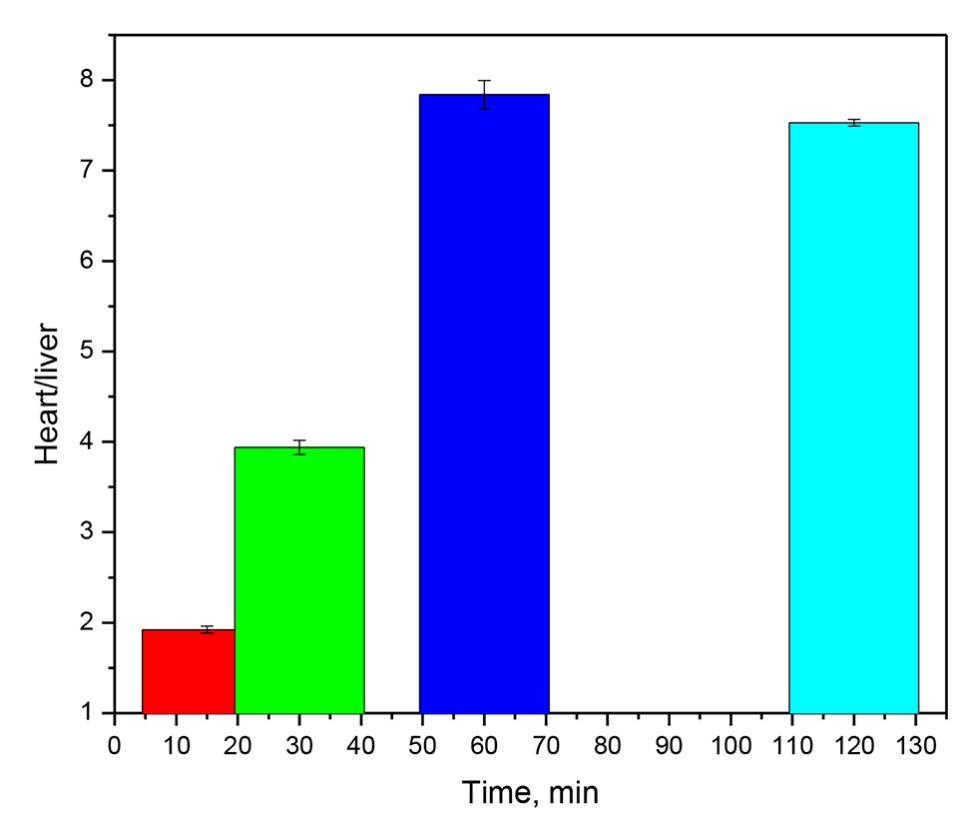
The ratio of heart to liver uptake ratio of [^131^I]iodocarvedilol with time

Table 2 shows the results, which are expressed as percent dose per gram of tissue of [^131^I]iodocarvedilol. The biological distribution of [^131^I]iodocarvedilol in rats revealed that it had a great myocardial uptake, good heart persistence, and minimal uptake in the liver, lungs and blood. It was quickly removed from non-target tissues. Both kidneys and liver were the primary excretion organs pathway. The ratio of heart to liver uptake was high (1.922, 3.94, 7.84, 7.84 and 7.53 at 5- 30-, 60-and 120-minute post-injection, respectively) (Fig. 8). The liver activity of [^131^I]iodocarvedilol was significantly decreased within the first hour compared with ^99m^Tc-Sestamibi activity which declined more slowly over time.

The target-to-non-target ratios of ^99m^Tc-sestamibi, a relatively successful myocardial imaging agent, are also presented in Table 3 [31] for comparison with [^131^I]iodocarvedilol.

[^131^I]iodocarvedilol had comparable heart to blood and heart to lung uptake ratios, but a significantly greater heart to liver uptake ratio, which is encouraging us to afford a novel myocardial imaging agent to get high-resolution scintigraphic images after using iodine-123 instead of iodine-131 (the same chemical properties).

**Table 2:** Biodistribution of [^131^I]iodocarvedilol in normal rats

Organs & Body fluids	% Injected dose/gram tissue at different time intervals, min			
	15	30	60	120
Blood	1.47 ± 0.13	0.21 ± 0.05	0.15 ± 0.02	0.20 ± 0.03
Bone	2.55 ± 0.17	1.43 ± 0.35	1.01 ± 0.21	0.8 ± 0.01
Muscle	4.17 ± 0.79	1.5 ± 0.04	1.2 ± 0.1	0.5 ± 0.02
Liver	12.74 ± 1.63	5.1 ± 0.15	2.5 ± 0.1	1.7 ± 0.1
Stomach	3.0 ± 0.03	2.6 ± 0.1	1.2 ± 0.16	1.1 ± 0.0
Intestine	6.4 ± 0.50	7.8 ± 0.3	8.8 ± 0.1	11.2 ± 0.03
Lung	5.5 ± 0.08	2.1 ± 0.12	1.3 ± 0.02	0.8 ± 0.01
Heart	24.5 ± 0.1	20.1 ± 0.3	19.6 ± 0.41	12.8 ± 0.25
Spleen	6.5 ± 0.31	2.9 ± 0.1	2.0 ± 0.02	1.7 ± 0.05
Kidney	30.2 ± 0.4	17.6 ± 0.6	10.6 ± 0.3	3.5 ± 0.06
Thyroid	1.1 ± 0.01	1.3 ± 0.01	1.2 ± 0.02	0.9 ± 0.05

Values represent mean ± SEM

**Table 3:** The ratios of target to non-target for [^131^I]iodocarvedilol and^99m^Tc-sestamibi in rats (n = 3)

Uptake ratio	[^131^I]iodocarvedilol			^99m^Tc-sestamibi			
	Post-injection time, min						
	5	30	60	5		30	60
Heart to liver	1.92	3.94	7.84	0.94	0.90		1.04
Heart to lung	4.46	9.57	15.1	2.87	9.07		12.25
Heart to blood	16.66	95.71	130.67	12.80	89.01		134.15

## Conclusion

In conclusion, using chloramine-T as an oxidizing agent, carvedilol was radiolabeled with NCA ^131^I by direct electrophilic substitution reaction with a high radiolabeling yield of 92.6 ± 2.77%. In rats, [^131^I]iodocarvedilol showed the right characteristics for use in cardiac imaging. It has a high heart uptake and heart to liver ratio, both of which are beneficial for high-quality SPECT myocardial imaging. [^131^I]iodocarvedilol solve most the drawbacks of the FDA approved ^99m^Tc-sestamibi.

## Data Availability

The datasets used and/or analyzed during the current study are available from the corresponding author on reasonable request.

## Abbreviations

CAD: Coronary artery disease
CAT: Chloramin-T
FDA: Food and Drug Administration
HPLC: High-performance liquid chromatography
ID: Injected dose
MPI: Myocardial perfusion imaging
Na/K-ATPase: Sodium-potassium adenosine triphosphatase
NCA: No-carrier-added
pi: Post injection
SPECT: Single-Photon Emission Computerized Tomography
T/B: Target-to-background ratio
TLC: Thin Layer Chromatography
